# The Role of Risk Perception in Students’ COVID-19 Vaccine Uptake: A Longitudinal Study

**DOI:** 10.3390/vaccines10010022

**Published:** 2021-12-24

**Authors:** Femke Hilverda, Manja Vollmann

**Affiliations:** Department of Socio-Medical Sciences, Erasmus School of Health Policy & Management, Erasmus University Rotterdam, 3000 DR Rotterdam, The Netherlands; vollmann@eshpm.eur.nl

**Keywords:** COVID-19, vaccine uptake, vaccination intention, risk perception

## Abstract

Since COVID-19 vaccine uptake was found to be especially low among young adults, the present study investigated COVID-19 risk perception as predictor of COVID-19 vaccination intention and actual COVID-19 vaccine uptake among this age group. More specifically, it was tested whether cognitive risk perception predicts vaccination uptake successively via affective risk perception and vaccination intention. In total, 680 students (65.9% female) between 17 and 28 years participated in this longitudinal online study. COVID-19 cognitive and affective risk perception, COVID-19 vaccination intention, and actual COVID-19 vaccine uptake were measured in t1: November/December 2020, t2: March 2021, and t3: June/July 2021, respectively. The mediation analysis revealed a significant indirect effect of perceived severity at t1 on vaccine uptake at t3 via worry at t1 and vaccination intention at t2. Stronger perceptions of perceived severity of COVID-19 were related to more worry about COVID-19, which led to a higher vaccination intention, which, in turn, increased the chance of COVID-19 vaccine uptake. To increase vaccine uptake among young adults it might be fruitful to emphasize the severity of COVID-19. However, one should take into account that tapping into fear works best when messages also include efficacy statements.

## 1. Introduction

Since the beginning of 2020, people worldwide have had to deal with the (consequences of the) outbreak of COVID-19, an illness caused by infection with the SARS-CoV-2 virus. The World Health Organization [1] declared COVID-19 a global pandemic on 30 January 2020. While several preventive measures, such as social distancing and increased hygiene rules, have been taken globally, the spread of COVID-19 is still ongoing. It has been suggested that vaccination is the most efficient way to prevent the coronavirus from further spreading and thus to stop the pandemic [2,3,4,5]. Vaccination contributes to reaching herd immunity, resulting in the indirect protection from infection caused by immunity of a large part of the population [6]. However, reaching herd immunity might be difficult, especially because immunity of individuals might be short-lived [7,8], and depends among other things on the efficacy of the vaccines [9] and virus mutation rates [10]. Even more important than herd immunity, vaccination lowers the severeness of COVID-19 symptoms, ultimately lowering the mortality of the disease [11,12,13]. Therefore, it is important to examine determinants of individuals’ COVID-19 vaccination intention and actual vaccine uptake.

A specific group of interest regarding COVID-19 vaccine uptake is young adults. The spread of the coronavirus is most prevalent among this group [14,15], while their vaccination intention and actual vaccine uptake is lowest compared to other age groups [3,16,17,18]. This makes it especially relevant to study determinants of vaccination intention and uptake in young adults. One possible determinant could be risk perception [19]. While multiple studies showed that risk perception is a predictor of the adherence to the COVID-19 behavioral guidelines [19,20,21,22] and COVID-19 vaccine acceptance [23,24,25], not much is known about the role of risk perception in actual COVID-19 vaccine uptake.

### 1.1. Risk Perception as Predictor of COVID-19 Protective Behaviors

Risk perception is a complex process, which is assumed to include both cognition as well as affect [26,27]. Cognitive risk perceptions are commonly divided into two dimensions, i.e., perceived susceptibility, which describes the perceived chance of getting a certain disease and perceived severity, which encompasses the perceived seriousness of the disease. In addition, affective risk perception, or sometimes called ‘affective response’, includes the feelings towards a certain risk people experience, mainly conceptualized as worry or anxiety. Cognitive and affective risk perception are related to each other and play an important role in predicting protective behaviors. One previous study regarding A/H1N1 influenza vaccination showed that cognitive risk perceptions influenced affective risk perception, which in turn predicted vaccination intention and subsequently vaccine uptake [28]. This result fits the ‘risk-as-feelings hypothesis’, which states that people’s feelings (partly) mediate the relation between cognitive risk perception and protective behaviors [29].

Regarding COVID-19, previous studies showed that both cognitive as well as affective risk perception stimulated adherence to the guidelines implemented to prevent the spread of COVID-19. That is, higher perceptions of chances to get infected with COVID-19 and higher perceived severity of COVID-19 were associated with a higher likelihood to implement protective behaviors [20,22,30,31,32]. Moreover, stricter adherence to the guidelines was related to affective risk perception, i.e., anxiety or worry about one’s own health [20,22,32] or the health of important others [20].

Previous studies also confirm the role of risk perception in COVID-19 vaccination intention [23,24]. One recent study showed that higher risk perception was related to more positive attitudes towards vaccine uptake [33]. Moreover, studies showed an association specifically between cognitive risk perception and intention to get vaccinated [25]. Additionally, a positive relationship between affective risk perception and vaccination intention was found. For example, Vollmann and Salewski [34] found that concerns about COVID-19 were related to a higher willingness to get vaccinated.

While previous research showed the importance of risk perception in COVID-19 vaccination intention, little is known about the role of both cognitive and affective risk perception in actual COVID-19 vaccine uptake. A recent systematic review [35], including 12 articles, found that cognitive risk perception predicted vaccine uptake. However, all studies included were cross-sectional. Our study adds to this by examining cognitive and affective risk perception as predictors of vaccination intention and subsequently vaccine uptake using a longitudinal design.

### 1.2. The Present Study

The present longitudinal study examined COVID-19 risk perception as a predictor of COVID-19 vaccination intention and vaccine uptake among young adults in the Netherlands. Cognitive and affective risk perception were measured before any COVID-19 vaccine was fully developed (first measurement point, t1: November/December 2020). Vaccination intentions were measured when COVID-19 vaccines were released, but not yet available for young adults (second measurement point, t2: March 2021). Actual vaccine uptake was measured when young adults had access to different COVID-19 vaccines (third measurement point, t3: June/July 2021).

**Figure 1:**
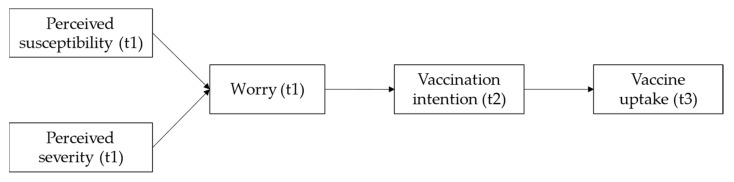
Graphic representation of the proposed mediating processes predicting COVID-19 vaccine uptake.

Following the model proposed by Renner and Reuter [28] and previous empirical findings, it was expected that cognitive risk perception (susceptibility and severity) about COVID-19 would predict actual COVID-19 vaccine uptake via affective risk perception (worry) about COVID-19 and COVID-19 vaccination intention. Figure 1 describes the predicted relationships between the concepts in this study.

## 2. Materials and Methods

### 2.1. Design and Procedure

This study is part of a larger research project examining university students’ experiences during the COVID-19 outbreak. A longitudinal cohort study was performed during the academic year 2020/2021 with three points of measurement (t1: November/December 2020, t2: March 2021, t3: June/July 2021) among Dutch university students. Students were included when they studied at a university that switched from offline teaching to online or blended teaching because of COVID-19 measures. Students who studied at an open university, studied parttime, or were aged above 30 years were excluded. The study was approved by the Medical Ethics Review Committee of the Erasmus Medical Center (#2020-0815).

Recruitment took place via student unions and student associations through email and social media posts, promoting a link to an online questionnaire in Qualtrics. After opening the link, students were informed about the purpose of the study and data handling. It was made explicit that participation was voluntary and anonymous, and that students could withdraw any time without any (negative) consequences. After providing informed consent, students could start filling in the questionnaire, which took them 20–30 min. Students who finished the questionnaire in less than 10 min were excluded from data analysis because this seemed unrealistic. Students received vouchers for participation (EUR 10, 15, and 25 for completing the questionnaire at t1, t2, and t3, respectively) to stimulate participation and prevent drop-out.

### 2.2. Measures

The online questionnaires measured several concepts regarding students’ experiences during COVID-19, such as life satisfaction, living circumstances, social interactions, study behavior, and learning outcomes. However, in this study, to test our hypothesized model, we only used a selection of variables. That is, we used background characteristics, risk perception measured at t1, vaccination intention measured at t2, and actual vaccine uptake measured at t3.

Risk perception was measured by numerical-cognitive estimates of the perceived susceptibility and severity of COVID-19. Based on Renner and Reuter [28], students indicated their absolute likelihood of becoming infected with COVID-19 and the severity of a COVID-19 infection on a 7-point scale ranging from 1 (very unlikely/not at all serious) to 7 (very likely/very serious). In addition, affective risk perception was operationalized as ‘worry’ using one adapted item of the Brief Illness Perception Questionnaire [36]. Students indicated how worried they were about COVID-19 on a 11-point scale ranging from 0 (not at all) to 10 (very much).

Vaccination intention was assessed with a single item adapted from Renner and Reuter [28]. Students indicated on a 7-point scale ranging from 1 (definitely not) to 7 (definitely yes) to what extent they were inclined to get vaccinated against COVID-19 once the vaccine is available to them.

Vaccine uptake was measured with one question adapted from Renner and Reuter [28]. Students were asked whether they were already vaccinated against COVID-19. Response options were ‘no’, ‘yes, partly’, and ‘yes, completely’. The two latter responses were merged, creating a dichotomous outcome measure with 0 = not vaccinated and 1 = vaccinated.

Background variables were measured with one item each. At t1, participants indicated their age and their gender (0 = male, 1 = female, 2 = nonbinary). Further, at all three measurement points, students indicated whether they had ever been infected with COVID-19 (0 = no, 1 = yes).

### 2.3. Data Analysis

Statistical analyses were performed with IBM SPSS Statistics Version 27 and Mplus version 8.5. Pearson correlations were calculated to investigate the bivariate associations between the study variables. As having been infected with COVID-19 was (marginally) significantly related to vaccination intention or vaccine uptake (see Table 1), it was included as control variables at each time point. The hypothesized sequential mediation model with the two dimensions of cognitive risk perception as predictors, affective risk perception and vaccination intention as sequential mediators, and vaccine uptake as outcome (controlling for having been infected at t1, t2, and t3) was tested by path analysis using logistic regression analysis based on maximum likelihood estimation. The indirect effects of the independent variables on the dependent variable via the mediator(s) were estimated by bootstrapping with 10,000 bootstrap samples as recommended by Hayes [37]. Coefficients are reported in standardized form.

## 3. Results

### 3.1. Participants

A total of 680 students participated in all three points of measurement and were used in the analyses. Details on participant flow are described in detail elsewhere [38]. Students of all 13 conventional universities and a variety of study fields took part. Both master students (30.8%) as well as bachelor students (69.2%) participated. A total of 65.9% of the participants was female, 33.4% was male, and 2 respondents identified as nonbinary. Age varied between 17 and 28 years, with a mean age of 21 years (*SD* = 2.06). Only a small part of the students had a migration background (6.5% Western migration background, 6.2% non-Western migration background). Most students lived in student housing or with friends (60.3%), followed by living with parents/family (26.2%), on their own (8.8%), with a partner (3.7%), or in another form (1.0%). The sample was representative for Dutch university students in terms of university affiliation, field of study, and study phase. However, males and students with migration background were underrepresented [39,40].

### 3.2. Bivariate Associations between Study Variables

The results of the correlation analyses (see Table 1) show that perceived severity, but not perceived susceptibility, at t1 was significantly positively related to worry at t1 and vaccination uptake at t3. Additionally, worry at t2 was (marginally) significantly positively associated with vaccination intention at t2 and vaccination uptake at t3. Finally, a significant positive correlation was found between vaccination intention at t2 and vaccination uptake at t3.

### 3.3. Prediction of COVID-19 Vaccine Uptake

**Figure 2:**
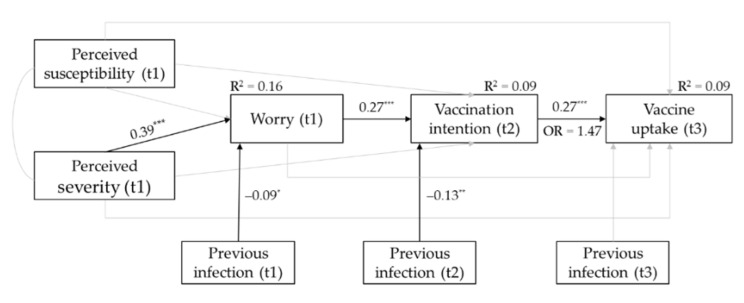
Results of the tested sequential mediation model. Grey paths were not statistically significant. * *p* < 0.05, ** *p* < 0.01, *** *p* < 0.001.

The results of the tested sequential mediation model are depicted in Figure 2.

Vaccine uptake at t3 was significantly predicted by vaccination intention at t2 with OR = 1.47, 95% CI [1.27, 1.72], indicating that for every one-unit increase in COVID-19 vaccination willingness at t2, the odds of being vaccinated against COVID-19 at t3 increased by 47%. Vaccination intention at t2 was significantly predicted by affective risk perception at t1, with more worry about COVID-19 leading to higher COVID-19 vaccination intention. Affective risk perception at t1 was significantly positively related to the cognitive risk perception dimension perceived severity at t1, with perceiving COVID-19 as more severe being associated with more worry about COVID-19.

Additionally, a significant positive indirect effect of perceived severity at t1 on vaccine uptake at t3 through worry at t1 and vaccination willingness at t2 was found, β = 0.03, BC 95% CI [0.017, 0.044]. This indirect effect indicates that stronger perceptions that a COVID-19 infection is severe are related to more worry about COVID-19, which leads to a higher COVID-19 vaccination intention, which, in turn, increases the chance of COVID-19 vaccine uptake.

## 4. Discussion

This study examined the role of both cognitive and affective risk perception in vaccination intention and subsequently vaccine uptake among students in the Netherlands using a longitudinal design with three points of measurement.

Our results showed that the intention to get vaccinated against COVID-19 is a significant predictor of actual COVID-19 vaccine uptake, confirming previous results from longitudinal studies regarding influenza vaccination [28,41]. Furthermore, affective COVID-19 risk perception was (marginally) related to COVID-19 vaccination intention as well as vaccine uptake, which is line with earlier research showing that worry about a disease promotes (the intention to) vaccine uptake [28,34,42,43].

For cognitive risk perception, significant positive associations of the dimension perceived severity of COVID-19 with worry about COVID-19 and to a lesser extent with COVID-19 vaccine uptake were found. This is largely in line with the assumption that cognitive risk perception informs affective risk perception [26,27,29] and with previous findings showing that perceived severity increases (COVID-19) vaccine acceptance [28,34,42,44]. Moreover, we found a significant indirect effect of perceived severity on COVID-19 vaccine uptake successively through worry and vaccination intention. Higher perceived severity of COVID-19 was associated with more worry about COVID-19 in November/December 2020, which had a favorable effect on vaccination intention in March 2021. In turn, vaccination intention positively predicted actual vaccine uptake in June/July 2021. These results partly confirm our hypothesized model and are in line with the ‘risk-as-feelings hypothesis’ [29] and previous studies [28,35]. In this way, our results show that risk perception does not only play a role in COVID-19 vaccination intention [23,24,25,34], but confirms the importance of risk perception in actual vaccine uptake [33,35].

In contrast, no significant effects were found for the cognitive risk perception dimension perceived susceptibility. That is, the perceived likelihood of getting infected with COVID-19 was neither related to worry about COVID-19 nor to COVID-19 vaccination intention and vaccine uptake. This is contrary to the expectations and previous research that found perceived susceptibility to be associated with more worry [28,45] and to be predictive of (the intention to) vaccine uptake [28,46,47,48]. However, another study among college students also found no associations between perceived susceptibility to COVID-19 and vaccine acceptance, while perceived severity was positively related to vaccine acceptance [45]. This implies that COVID-19 vaccine acceptance and uptake among young adults is related to the perceived severity of COVID-19 rather than the perceived chance of getting it. One reason for this might be that while COVID-19 vaccination reduces the severity of symptoms and number of deaths [49], it does not automatically prevent infection because immunity may wane over time [50]. Incidence of COVID-19 among young adults is high, while the burden of disease is relatively low [51]. This may explain why susceptibility alone does not cause worry and hence does not stimulate vaccine uptake. Similar results were found in a study conducted in China about seasonal influenza vaccination. That is, perceived susceptibility was not associated with vaccine uptake among young adults but was a significant predictor for older adults [52]. For young adults, it is thus important to focus on the possible serious consequences of COVID-19 and emphasize that vaccination can lower the seriousness of the infection. Regarding diseases with a high burden of diseases among young adults, perceived susceptibility might be an important predictor of vaccination.

### 4.1. Practical Implications

Our results showed that stronger perceptions of perceived severity of COVID-19 are related to more worry about COVID-19, which leads to a higher vaccination willingness, which, in turn, increases the chance of COVID-19 vaccine uptake. This finding indicates that emphasizing possible severe consequences of COVID-19 might be a reasonable approach to increase the vaccination rate among young adults. Another study found that highlighting the consequences for older vulnerable others might help young adults to adhere to the preventive guidelines [20]. In a similar way, young adults might be motivated to get vaccinated if the severity of COVID-19 is pointed out for themselves, but also when the severity of COVID-19 for vulnerable others is explained. Moreover, while the mortality of young adults with COVID-19 is low [51], more and more cases in which young adults suffer from severe and long-term symptoms are found [53]. Vaccination will help to lower the severity of the symptoms and decrease mortality [49].

While communicating about the risk of COVID-19 might be important to stimulate young adults to get vaccinated, it is also key to address efficacy aspects in risk messages [54]. That is, inducing worry or fear by emphasizing severity of COVID-19 is only useful if individuals are given an action perspective. In this case, young adults need to know where and how they can sign up for vaccination. To persuade young adults to partake in the vaccination program, different strategies might be used. For example, role models, such as social media influencers, might stimulate their peers to get themselves vaccinated [55] or experience experts (i.e., young adults who suffered from COVID-19) might be used as spokespersons to inform young adults about the possible consequences of getting infected with COVID-19 and the necessity of the vaccine.

### 4.2. Strengths, Limitations, and Future Research

This study provided unique insights into the role of risk perception in COVID-19 vaccine uptake. One key aspect was the longitudinal design used. This design allowed us to examine effects of risk perception and intention on actual vaccine uptake over time. Moreover, we operationalized cognitive risk perception differentiating between severity and susceptibility, enabling us to pinpoint the contribution of cognitive risk perception in COVID-19 vaccine uptake more precisely, showing that perceived severity is more relevant than perceived susceptibility in the case of COVID-19.

However, some limitations of this study need to be considered. Firstly, while this study provides in-depth insight into the role of risk perception in vaccination, it did not include other possible determinants. Our model explained 9% of vaccine uptake, suggesting that while risk perception is a significant determinant of vaccine uptake, other perceptions might also play a role. For example, vaccine related perceptions such as necessity beliefs and concerns about side effects, may be other relevant determinants [34,35]. Therefore, we can only make conclusions about the role of risk perception on its own, but not relate this to or compare its effect with the effects of other determinants. Secondly, as this study focused on university students, we cannot generalize to other age groups or education levels. It thus remains uncertain if our tested model also applies to older individuals and young adults with lower education levels.

Future research would benefit from including a diverse sample in terms of age and education level. In addition, a more complex model including multiple determinants might be tested in further studies.

## 5. Conclusions

To stimulate young adults to get vaccinated against COVID-19, hereby increasing vaccine uptake and possibly stopping the spread of COVID-19, it might be fruitful to emphasize the severity of COVID-19 among this age group. Stronger perceptions of perceived severity of COVID-19 are related to more worry. Tapping into fear or worry, preferably combined with efficacy statements, may induce the intention to get vaccinated, which in turn may stimulate vaccine uptake.

## Figures and Tables

**Table 1:** Descriptives and Pearson correlations between study variables.

	1	2	3	4	5	M (SD)	% ^e^
1. Perceived susceptibility t1 ^a^						4.41 (1.45)	
2. Perceived severity t1 ^a^	0.03					2.59 (1.34)	
3. Worry t1 ^b^	–0.03	0.39 ***				4.67 (2.50)	
4. Vaccination intention t2 ^a^	0.03	0.05	0.25 ***			6.27 (1.36)	
5. Vaccine uptake t3 ^c^	0.04	0.08 *	0.07 ^(^*^)^	0.23 ***			
6. Previous infection t1 ^c^	0.29 ***	–0.04	–0.11 **	–0.11 **	0.02		23.2
7. Previous infection t2 ^c^	–	–	–	–0.12 **	–0.02		28.2
8. Previous infection t3 ^c^	–	–	–	–	–0.08 ^(^*^)^		31.3
9. Age	0.06	0.10 **	0.01	0.02	0.04	21.01 (2.06)	
10. Gender ^d^	0.03	0.15 **	0.15 **	–0.04	0.00		66.1

Note. ^a^ scale range 1–7; ^b^ scale range 0–10; ^c^ dichotomous 0 = no, 1 = yes; ^d^ dichotomous 0 = male, 1 = female, the two nonbinary students were excluded; ^e^ percentage of code 1. Bivariate associations including one or two dichotomous variables were also investigated with *t*-tests and χ^2^-tests, respectively. These analyses revealed the same results as the Pearson correlation analyses. ^(^*^)^ *p* < 0.10, * *p* < 0.05, ** *p* < 0.01, *** *p* < 0.001.

## Data Availability

Data used in this study are made publicly available in the Erasmus University Repository. https://doi.org/10.25397/eur.17182292 (accessed on 29 November 2021).
